# Epigenetics in glaucoma: a link between DNA methylation and neurodegeneration

**DOI:** 10.1172/JCI163670

**Published:** 2022-11-01

**Authors:** Wendy W. Liu, Yang Sun

**Affiliations:** 1 Department of Ophthalmology, Stanford University School of Medicine, Palo Alto, California, USA.; 2 Palo Alto Veterans Administration, Palo Alto, California, USA.

## Abstract

Normal-tension glaucoma is a form of optic nerve degeneration that is characterized by loss of retinal ganglion cells independent of eye pressure elevation. In this issue of the *JCI*, Pan et al. report the discovery in a Japanese family of a mutation in the *METTL23* gene, which encodes a DNA methyltransferase that causes normal-pressure glaucoma in haploinsufficiency. Inherited as an autosomal dominant condition, *METTL23* deficiency revealed an important function in the regulation of pS2 and the downstream NF-κB signaling pathway, which has previously been linked to glaucomatous optic nerve degeneration. These findings are the first direct link between defective epigenetic regulatory machinery and genetic forms of optic nerve degeneration.

## Black cataract

Ancient Indian medical culture described glaucoma as “black cataract”— the loss of vision that leads to blindness in the absence of clouding of the lens. The term glaucoma has its origin in the Greek word glaukos, meaning “shiny” or “grayish blue.” This term is thought to describe the bluish pupillary hue found in some affected eyes (1). In the mid-19th century, improved methods for measuring intraocular pressure (IOP) and the invention of the ophthalmoscope allowed for better descriptions of glaucoma as a clinical entity. Soon after, glaucoma was proposed to be a pressure excavation, as elevated IOP and excavation of the optic nerve were discovered as key features that distinguished glaucoma from other ophthalmologic conditions (2). Today, glaucoma is the leading cause of irreversible blindness, affecting approximately 80 million people worldwide. IOP is a major risk factor for most types of glaucoma. Elevated IOP is thought to be the mechanical force that drives the damage of retinal ganglion cells, leading to the clinical features of optic nerve excavation and visual field loss. For the past century, lowering the IOP has been the mainstay of glaucoma treatment, with much research effort directed at understanding how IOP affects retinal ganglion cell (RGC) survival (3).

## Glaucoma with normal pressure

Although the term glaucoma is historically associated with high IOP, a substantial proportion of patients with glaucoma do not have elevated IOP. They have features of optic nerve excavation and visual field loss characteristic of glaucoma, but their IOP is within the normal range. The underlying pathophysiology of this kind of glaucoma is not well understood and is referred to as “normal-tension glaucoma” (NTG) (4). IOP has been shown to be a contributing factor for NTG, and reducing IOP slows the rate of glaucoma progression in patients with NTG (5). In addition, vascular dysfunction and poor optic nerve head perfusion have been proposed as important pathogenic factors. In Japan, the vast majority of patients with glaucoma fall into the NTG category (6). In the United States, the prevalence of NTG is thought to be as high as 30% to 39%. This observation raises interesting questions. If mechanical forces from high IOP lead to ganglion cell loss in glaucoma, why do patients with normal IOP also develop optic nerve excavation and field loss? In addition to IOP, what other factors and cellular pathways are involved in glaucoma pathogenesis?

The increasing recognition that eye pressure–independent forms of glaucoma are contributing to blindness worldwide has broadened the search for genes that are intrinsic to RGC survival. In recent years, genetic and genomic studies have uncovered many genes contributing to glaucoma (7). Mendelian inheritance is common for rare, early-onset forms of the disease, while adult-onset forms that are more prevalent typically have complex inheritance. Discovery of these genes and genetic loci provides insight into the molecular mechanisms of the disease and forms the basis for developing gene-based therapies and diagnostics. Genes that have been implicated in glaucoma include those involved in diverse cellular processes, such as inflammatory responses, lipid metabolism, ocular development, and mitochondrial function. To date, rare mutations in two genes have been found to cause early-onset familial NTG with autosomal dominant inheritance: optineurin (*OPTN*) and Tank-binding protein 1 (*TBK1*). *OPTN* and *TBK1* are known to interact and have critical roles in autophagy and the NF-κB signaling pathways. These genes account for approximately 2% to 3% of NTG cases. Hence, additional genes involved in the pathogenesis of NTG have yet to be discovered. The discovery of these genes will not only shed light on the pathogenesis of glaucoma, but may also inform our understanding of other neurodegenerative diseases.

## Epigenetics and neurodegeneration

**Figure 1:**
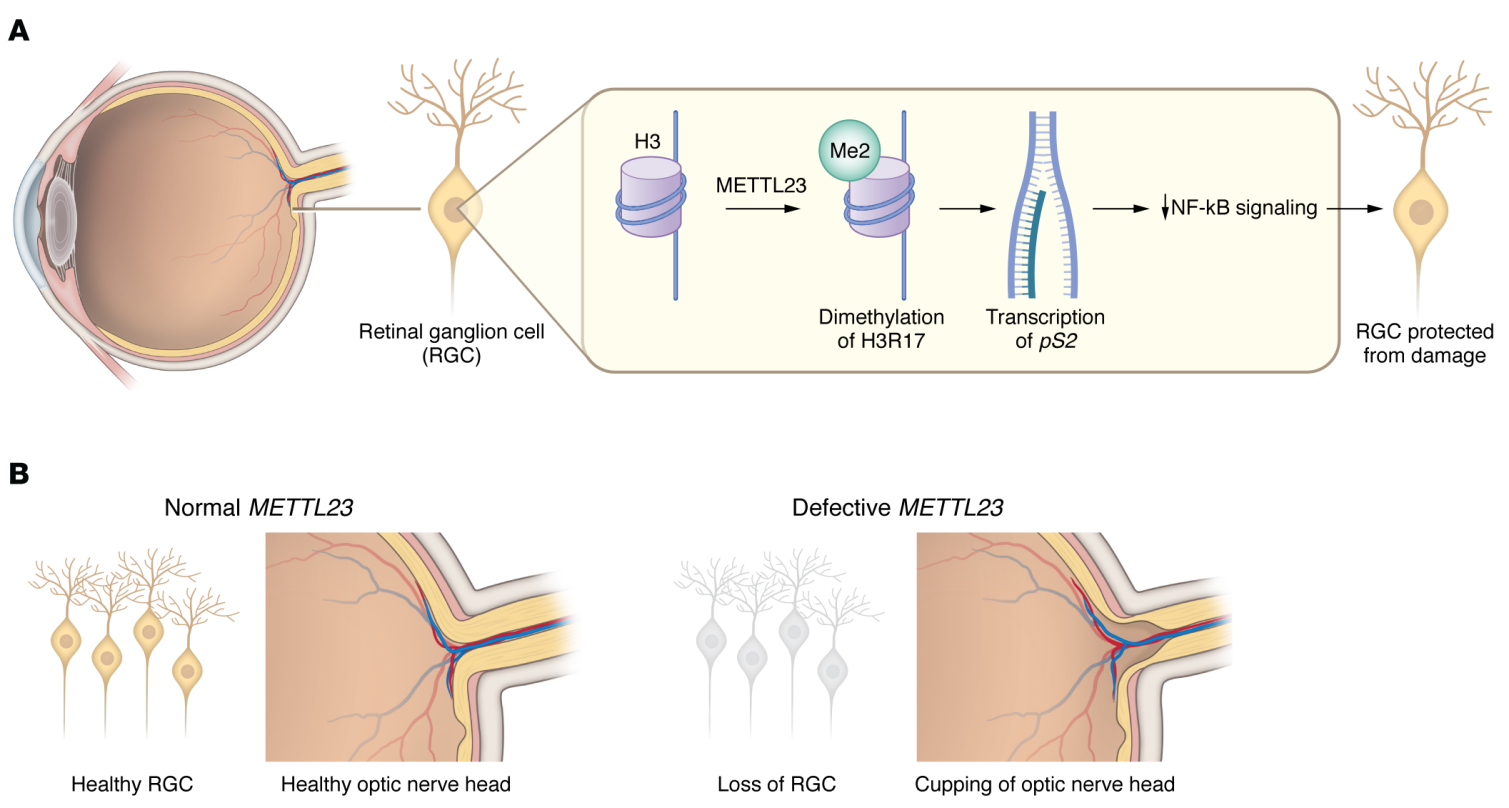
A model for the role of METTL23 in RGC survival and optic nerve neuroprotection. METTL23 catalyzes the dimethylation of H3R17 in the retina. Its activity ensures transcription of the estrogen receptor *pS2*, which negatively regulates NF-κB signaling and protects RGCs from glaucomatous damage. *METTL23* loss of function results in RGC death and normal-pressure glaucoma.

The elegant study by Pan et al. in this issue of the *JCI* describes a genetic cause of NTG and reports a mutant histone methyltransferase leading to NTG (8). The investigators identified a family of three generations of Japanese patients with NTG and a splicing mutation in the methyltransferase-like 23 (*METTL23*) gene, which encodes a histone arginine methyltransferase. The autosomal dominant pattern of inheritance supporting the *METTL23* c.A83G mutation was found in all six affected members of the family. This mutation resulted in *METTL23* mRNA aberrant splicing. The haploinsufficiency of this gene resulted in decreased levels and abnormal subcellular localization of protein. Mechanistically, METTL23 catalyzes dimethylation of H3R17 in the retina. The estrogen receptor pS2 was identified as a target effector of this methylation activity and negatively regulated NF-κB signaling (Figure 1). Using multiple approaches, including *Mettl23*-knockin and -knockout mice and NTG patient–derived induced pluripotent stem cells (iPSCs), the investigators showed a critical function of *METTL23* in RGC soma survival and optic nerve neuroprotection. Nonetheless, there may be other factors that modulate the phenotype of *METTL23* heterozygosity. A *METTL23 c.84+60delAT* variant that promotes exon 2 skipping was enriched in patients with NTG but could also be found in controls (1.4% of patients with NTG and 0.6% of controls), suggesting that *METTL23* splice variants may have varying pathogenicity.

A notable contribution of Pan et al. (8) is the link between histone methylation and glaucoma, which may have implications for other forms of neurodegenerative disorders. There has been increasing evidence that alterations in DNA methylation play an important role in neurodegenerative diseases, including Alzheimer’s disease, Parkinson’s disease, and amyloid lateral sclerosis (ALS) (9). Interestingly, both *OPTN* and *TBK1* mutations have been identified in patients with ALS (10). PRMT1-mediated arginine methylation of RNA-binding protein fused in sarcoma (FUS), also implicated in ALS, causes aggregates in the cytoplasm that may inhibit FUS function in RNA splicing, DNA repair, and transcriptional regulation that are essential for neuronal homeostasis (11).

Another major epigenetic regulatory mechanism revolves around histone acetylation and methylation. The enzymes involved in these processes have also been linked to RGC survival in animal models of optic nerve injury and neurodegenerative diseases (12, 13). RGC-specific knockout of histone deacetylase (Hdac3) resulted in reduced heterochromatic formation and increased RGC survival in the optic nerve crush model of optic neuropathy (12). However, human variants in HDACs have not been associated with glaucoma.

This study by Pan et al. highlights the role of histone arginine methylation in neurodegenerative processes and raises important questions for the field (8). Are there other targets of *METTL23* that play critical roles in RGC survival? Do other pathways downstream of *METTL23* mediate its protective effect in RGCs? Are *METTL23* mutations observed in other neurodegenerative conditions? NF-κB–mediated inflammation has been suggested to play a role in NTG and other neurodegenerations. However, its pleiotropic effects in the body may make it a challenging target in the design of neuroprotective strategies. Will *METTL23* be a more viable target? The study by Pan and colleagues (8) opens new areas of epigenetics as therapeutic targets for glaucomatous neurodegeneration and provides important evidence for the role of histone arginine methylation in neuronal survival and function.

## Version 1 11/01/2022

Electronic publication
